# Identification of Loci That Confer Resistance to Bacterial and Fungal Diseases of Maize

**DOI:** 10.1534/g3.120.401104

**Published:** 2020-06-22

**Authors:** Yuting Qiu, Julian Cooper, Christopher Kaiser, Randall Wisser, Santiago X. Mideros, Tiffany M. Jamann

**Affiliations:** * Dept. of Crop Sciences, University of Illinois at Urbana-Champaign, Urbana, IL 61801 and; ^†^ Dept. of Plant and Soil Sciences, University of Delaware, Newark, DE 19716

**Keywords:** Multiple disease resistance, maize, Goss’s wilt, quantitative disease resistance, Genetics of Immunity

## Abstract

Crops are hosts to numerous plant pathogenic microorganisms. Maize has several major disease issues; thus, breeding multiple disease resistant (MDR) varieties is critical. While the genetic basis of resistance to multiple fungal pathogens has been studied in maize, less is known about the relationship between fungal and bacterial resistance. In this study, we evaluated a disease resistance introgression line (DRIL) population for the foliar disease Goss’s bacterial wilt and blight (GW) and conducted quantitative trait locus (QTL) mapping. We identified a total of ten QTL across multiple environments. We then combined our GW data with data on four additional foliar diseases (northern corn leaf blight, southern corn leaf blight, gray leaf spot, and bacterial leaf streak) and conducted multivariate analysis to identify regions conferring resistance to multiple diseases. We identified 20 chromosomal bins with putative multiple disease effects. We examined the five chromosomal regions (bins 1.05, 3.04, 4.06, 8.03, and 9.02) with the strongest statistical support. By examining how each haplotype effected each disease, we identified several regions associated with increased resistance to multiple diseases and three regions associated with opposite effects for bacterial and fungal diseases. In summary, we identified several promising candidate regions for multiple disease resistance in maize and specific DRILs to expedite interrogation.

Plants need to defend themselves from many pathogenic microbes present in their environment. Furthermore, the widespread cultivation of varieties with limited genetic diversity increases the risk of pathogen attack (Strange and Scott 2005). Crops are seldom attacked by just a single pathogen, and thus, breeding is usually conducted for resistance to multiple pathogens (Khush 1989; Ceballos *et al.* 1991). Multiple disease resistance (MDR) is defined as host plant resistance to more than one disease and is controlled by single to many genes (Wiesner-Hanks and Nelson 2016; Nene 1988). Despite the widespread need across many crops for multiple disease resistant varieties, little is known about the genetic determinants of MDR. A few cloned disease resistance quantitative trait loci (QTL) have been shown to provide protection against multiple diseases including *Lr34* and *Lr67* in wheat (Krattinger *et al.* 2009; Moore *et al.* 2015) and *GH3-2* in rice (Fu *et al.* 2011). Genes conferring resistance to multiple diseases include those that encode signaling pathways, pathogen recognition, hormone-associated defense initiation, antimicrobial peptides, sugar signaling and partitioning pathways, and cell death-related pathways (Wiesner-Hanks and Nelson 2016). A more thorough understanding of MDR in crops will facilitate the development of varieties resistant to multiple diseases.

Maize is a staple cereal affected by over 32 major diseases that can cause substantial yield losses (Mueller *et al.* 2016; Munkvold and White 2016). Foliar diseases can cause significant production constraints, particularly in conducive environments. A survey from 2012 to 2015 showed that foliar diseases of maize lead to the largest estimated yield losses in the northern U.S. corn belt in non-drought years (Mueller *et al.* 2016). Pesticides are available to manage fungal foliar diseases but are costly and have environmental impacts (Paul *et al.* 2011; Bartlett *et al.* 2002). No labeled effective chemical control is available for the major bacterial foliar diseases. An effective and environmentally benign method of disease management is host plant resistance (Nelson *et al.* 2018). The heritability for foliar diseases are moderate to high, indicating breeding to develop resistant varieties is possible (Dingerdissen *et al.* 1996; Lopez-Zuniga *et al.* 2019; Zwonitzer *et al.* 2010; Ceballos *et al.* 1991).

Many MDR mapping studies in maize have focused on fungal diseases, and less is known about the relationship between resistance to fungal and bacterial diseases. In a synthesis study Wisser *et al.* (2006) examined the relationship between fungal, bacterial, and viral resistance and identified loci that conferred resistance to fungal and bacterial diseases. Subsequent studies identified regions, and even genes, that confer resistance to the three most significant fungal foliar diseases – southern corn leaf blight (SCLB), northern corn leaf blight (NCLB), and gray leaf spot (GLS) (Lopez-Zuniga *et al.* 2019; Zwonitzer *et al.* 2010; Belcher *et al.* 2012; Yang *et al.* 2017b). Relatively few regions have been identified that confer resistance to both a fungal and a bacterial pathogen in maize (Chung *et al.* 2010; Chung *et al.* 2011; Qiu *et al.* 2020; Jamann *et al.* 2014; Jamann *et al.* 2016; Hu *et al.* 2018). In this study, we focused on two bacterial diseases bacterial leaf streak (BLS) and Goss’s bacterial wilt and blight (GW), as well as three fungal diseases: SCLB, NCLB, and GLS.

Goss’s wilt and bacterial blight is one of the most destructive foliar diseases of maize (Mueller *et al.* 2016) and is caused by *Clavibacter nebraskensis* (Li *et al.* 2018). The blight phase of the disease is characterized by water-soaked tan to gray linear lesions with irregular margins parallel to, but not bounded by, leaf veins. The bacteria colonize the xylem, and vascular wilt symptoms can develop in susceptible lines (Mbofung *et al.* 2016; Jackson *et al.* 2007; Schuster 1972). The bacteria usually enter the leaves through wounds, but can also enter through natural openings in the absence of wounding in high-humidity conditions (Mallowa *et al.* 2016). First identified in 1969, GW is now found throughout the midwestern United States and Canada (Malvick *et al.* 2010; Howard *et al.* 2015; Singh *et al.* 2015; Schuster 1972; Mueller *et al.* 2016; Jackson *et al.* 2007).

Bacterial leaf streak, caused by *Xanthomonas vasicola* pv. *vasculorum* (*Xvv*), is an emerging disease in the Americas (Damicone *et al.* 2018; Jamann *et al.* 2019; Korus *et al.* 2017; Leite *et al.* 2019). The bacteria enter and exit through wounds and stomata to colonize intercellular spaces, but do not enter the vasculature (Ortiz-Castro *et al.* 2018). NCLB, GLS, and SCLB are among the most important fungal foliar diseases. NCLB is of global importance and is caused by the hemibiotrophic pathogen *Setosphaeria turcica* (syn. *Exserohilum turcicum*). In inoculated trials using susceptible germplasm, NCLB caused a 30–62% grain yield reduction (Perkins and Pedersen 1987; Raymundo and Hooker 1981). Humid conditions and moderate temperatures favor NCLB development. Gray leaf spot is also of global importance and is caused by the necrotrophic fungi *Cercospora zeae-maydis* and *Cercospora zeina*. Gray leaf spot can cause as much as a 50% yield loss (Ward *et al.* 1999) and develops quickly in high humidity conditions. Southern corn leaf blight, caused by *Bipolaris maydis*, is usually found in hot and humid regions and can cause up to a 40% yield loss if the varieties are susceptible, and the conditions are favorable (Byrnes *et al.* 1989). All the diseases are favored by high humidity environments. There are overlapping pathogenesis and tissue-level pathogen localization between diseases. For example, the pathogens causing NCLB and GW both colonize the xylem (Chung *et al.* 2010; Mbofung *et al.* 2016), and for both BLS and GLS the pathogen enters the stomata (Beckman and Payne 1982; Ortiz-Castro *et al.* 2018).

We conducted linkage mapping for GW in a chromsome segment substitution line (CSSL) population, referred to as a disease resistance introgression line (DRIL) population. We selected a DRIL population because it was developed to study multiple disease resistance (Lopez-Zuniga *et al.* 2019). Data for BLS (Qiu *et al.* 2020), SCLB, NCLB, and GLS (Lopez-Zuniga *et al.* 2019) were combined with the GW data to examine MDR. We evaluated the DRIL78 population, an ideal population for this study, as the donor line NC344 is resistant and the recurrent parent Oh7B is susceptible for all the diseases studied (Cooper *et al.* 2019; Qiu *et al.* 2020; Lopez-Zuniga *et al.* 2019). Thus, we hypothesized that we could identify regions for resistance to fungal and bacterial pathogens in this population.

Multivariate analysis was used to identify potential MDR loci. Multivariate analysis based on Mahalanobis distance (Md) has been used for genome scans in both human and plant studies (Luu *et al.* 2017; Tian *et al.* 2008; Lotterhos *et al.* 2017). In this study, we used Md to combine the mapping results from the five diseases. Md is not trait-specific; instead, it is a test for outlier markers across all traits and takes multiple mapping result datasets into consideration. The outlier markers, reported as putative MDR markers, are those that do not follow the pattern of the majority of the data point cloud (Rousseeuw and Van Zomeren 1990).

The overall objective of this study was to compare the genomic basis of resistance to fungal and bacterial diseases in maize. Mapping was conducted for GW using phenotypic data collected in three environments and combined with previously published studies for BLS, NCLB, SCLB, and GLS (Lopez-Zuniga *et al.* 2019; Qiu *et al.* 2020). Here, we: 1) identify novel QTL associated with GW through linkage mapping; 2) explore the relationship between the five diseases in this population; and 3) estimate the effect of potential MDR haplotypes on the five diseases.

## Materials and Methods

### Plant materials

Disease resistance introgression line population DRIL78 is an ideal CSSL population for multiple disease evaluation, as the donor parent (NC344) is multiple disease resistant and the recurrent parent (Oh7B) is multiple disease susceptible (Lopez-Zuniga *et al.* 2019; Cooper *et al.* 2019; Qiu *et al.* 2020; Wisser *et al.* 2011). The population was developed by a cross between NC344 and Oh7B, three generations of backcrosses, and four subsequent generations of self-pollinating via single-seed descent to obtain BC_3_F_4:5_ lines (Lopez-Zuniga *et al.* 2019). This population was selected because preliminary data showed significant differences between the parents of this population for all diseases examined.

### Phenotypic evaluation

The DRIL78 population was evaluated in three environments: Urbana 2016, Monmouth 2017, and Urbana 2017. The Urbana trials were conducted at the University of Illinois Crop Science Research and Education Center South Farms located in Urbana, IL. The Monmouth trial was conducted at the University of Illinois Monmouth Research Station located in Monmouth, IL. In Urbana 2016, 260 lines were evaluated for GW in one replication. In 2017, 229 and 233 lines were evaluated in Monmouth and Urbana, respectively, each with two replications. Differences in the number of lines evaluated was due to seed availability and not relative to disease phenotype. For Monmouth and Urbana 2017, we generated an incomplete block design using the agricolae package in R (Version 3.5.1) (de Mendiburu and de Mendiburu 2019; R Core Team 2018). For Monmouth 2017 and Urbana 2017, Oh7B was included in each block, along with the resistant check line NC344 or NC258. NC344 was not included in every block due to seed availability. For Urbana 2016, we used an augmented incomplete block design with one replication. In this location, the parental lines NC344 and Oh7B were included in each block. Seed was machine planted with 20 kernels per plot. Plots were 3.2 meters with 0.76 m alleys between each plot and row spacing of 0.762 meters. Fields were managed using standard agronomic practices for central Illinois.

### Disease evaluation

*Clavibacter nebraskensis* isolate 16Cmn001 was used for the GW inoculations (Cooper *et al.* 2018). We inoculated the plants twice, once at the V4 stage and a second time at the V7 stage (Abendroth *et al.* 2011), as described by Cooper *et al.* (2018). Two inoculations improved the differentiation between lines. We assessed the extent of necrosis of inoculated plants using a visual percentage rating on a per plot basis with 5% intervals starting about two weeks after the first inoculation date. A rating of 0% represented no disease in the plot, while 100% indicated that all the foliage was necrotic (Poland and Nelson 2011). In Urbana 2016, two visual ratings were taken 17 days apart; in Urbana 2017, two ratings were taken 18 days apart; in Monmouth 2017, three ratings were taken with 8 and 9 days between ratings. We calculated the area under the disease progress curve (AUDPC) scores for each plot in R (Version 3.5.1) (R Core Team 2018) using the *audpc* function in the agricolae package (de Mendiburu and de Mendiburu 2019) (File S1).

### Statistical analysis

Least Square Means (LSMeans) were estimated for GW for each environment (2016 Urbana, 2017 Urbana, and 2017 Monmouth) and for the combined multienvironment dataset using AUDPC values and the *lmer* function in the R package lme4 (Doran *et al.* 2007). Linear mixed models were constructed for each environment and the combined dataset and are listed below:

Urbana 2016: ${\text{Y}}_{\text{ijk}}=\mu +G_{i}+B_{j}+\epsilon_{ijk}$;
Urbana 2017, Monmouth 2017: ${\text{Y}}_{\text{ijkl}}=\mu +G_{i}+R_{j}+B{\left(R\right)}_{\left(j\right)k}+\epsilon_{ijkl}$;
Combined dataset: ${\text{Y}}_{\text{ijklm}}=\mu +G_{i}+E_{j}+GE_{ij}+R{\left(E\right)}_{\left(j\right)k}+B{\left(R\left(E\right)\right)}_{\left(jk\right)l}+\epsilon_{ijklm}$;

where Y is the response variable (AUDPC) as described above, *μ* is the overall mean, *G* is the fixed genotype (introgression line) effect, *B* is the random blocking effect, *R* is the random replication effect, *E* is the random environment effect, and *GE* is the random genotype-by-environment interaction effect. Blocks were nested within replications within environments. Only significant factors were included in the models. We examined the skewness of the data using the *skewness* function from the e1071 package (Dimitriadou *et al.* 2009). Heritability on both a plot and family-means basis were calculated for GW with SAS (version 9.4) using PROC MIXED, as described by Holland *et al.* (2003).

We calculated LSMeans for the BLS data based on the raw measurements from Qiu *et al.* (2020). The model included genotype as a fixed factor, and replication and block nested within replication as random factors. We obtained LSMeans for SCLB, NCLB, and GLS from Lopez-Zuniga *et al.* (2019).

Multiple comparison tests were conducted using the LSMeans calculated for each disease individually to identify the lines that were significantly different from the recurrent parent Oh7B. To perform the tests we used the package multcomp in R, specifically the function *glht*, with a Dunnett’s *p*-value adjustment (Hothorn *et al.* 2016).

### Disease correlations

We conducted Pearson’s product-moment correlation tests among LSMeans for the diseases (ten total comparisons) in R using the *cor.test* function. The parent lines were not included. SCLB, NCLB, and GLS were rated using a 1-9 rating scale, where 1 indicated 100% leaf area affected by the pathogen and 9 indicated no disease; BLS phenotypes were lesion length measurements where small values indicated shorter lesions; GW was rated using a percentage scale based on the severity of the disease where 0% indicated no disease. To have a uniform scale for correlation analysis, we multiplied the BLS and GW LSMeans values by -1. With this modification, low values indicated more severe infections for all datasets.

### Linkage mapping

A total of 190 lines, including the recurrent parent Oh7B, were shared across all five datasets. We used the LSMeans for 190 lines and 237 single nucleotide markers from Lopez-Zuniga *et al.* (2019) to conduct linkage mapping for each of the five diseases (File S2). The software ICIMapping 4.0.6.0 with the options “CSL” and “RSTEP-LRT-ADD” mapping were used to conduct QTL analysis (Meng *et al.* 2015). We conducted 1000 permutations with a 0.10 Type I error rate to determine the logarithm of odds (LOD) threshold. We recalculated the LOD threshold for each disease. The physical positions of markers with LOD values exceeding the threshold are reported based on B73 RefGen_v3 coordinates (Schnable *et al.* 2009).

### Multivariate analysis

We conducted multivariate analysis to identify QTL associated with more than one disease using the methods described in Lopez-Zuniga *et al.* (2019). The five diseases each served as a variable and the “robust Mahalanobis distance” method was used to combine the five variates to detect outlier markers. In this study, Mahalanobis distance (Md) was calculated based on the five negative log10 *p*-values of the LOD scores derived from the five single-disease mapping results. Outlier markers were detected based on *p*-values for Md. The detailed steps of multivariate analysis are described below: (i) conduct linkage mapping analysis with ICIMaping for each trait in the population independently; (ii) obtain trait-specific, permutation-based LOD thresholds and trait-specific marker LOD values from the mapping results; (iii) calculate *p*-values for each marker for each disease based on the following function:

$$\text{P}\left(\text{LOD}\right)=0.5\times (\chi_{1}^{2}>2ln10\times LOD)$$

to account for the variation in LOD significance thresholds between different mapping studies (Nyholt 2000); (iv) convert *p*-values into negative log10 *p*-values; (v) calculate Mahalanobis distance based on negative log *p*-values (Md-p) for each of the diseases in R with *OutlierMahdist* function in rrcovHD package (Todorov 2018), as described by Lotterhos *et al.* (2017); (vi) calculate *p*-values for Md-p for each marker (Rousseeuw and Van Zomeren 1990). To control for multiple comparisons, the false discovery rate (FDR) was calculated by adjusing the *p*-values using the “BH” method (Hochberg and Benjamini 1990) with the *p.adjust* function in R. Markers were declared to be significant using a 1% FDR.

### Haplotype effect calculation

The maize genome has previously been divided into 100 bins which we used here to delineate disease resistance-associated segments of the genome (Davis *et al.* 1999). The chromosomal bin for each marker that passed the 1% FDR Md-p test and the single-disease linkage mapping analysis was recorded. We considered bins with at least three significant Md-p markers as candidate MDR regions. The selected MDR regions were delimited by the position of the two flanking significant markers. To calculate the haplotype effect for each region, we identified lines with introgressions in the MDR regions and then calculated, using the raw AUDPC data, the difference between the mean AUDPC for those lines and the mean AUDPC for the recurrent parent Oh7B (Belcher *et al.* 2012). Because different scales were used for each disease and we wanted to compare between diseases, we standardized the haplotype effect by Oh7B.

$$\text{Percentage change}=\frac{\text{Haplotype effect}}{\mu_{Oh7B}}\times 100\text{\%}$$

Finally, we conducted a *t*-test using the percentage change to determine whether there was a significant difference between the Oh7B phenotype and the introgression line effect. The null hypothesis was that there is no difference between Oh7B and the haplotype effect (percent change = 0).

### Data availability

File S1 contains the AUDPC values for Goss’s wilt for all evalauated lines. File S2 contains the genotypes and LSMeans used for ICIMapping. Data from Lopez-Zuniga *et al.* (2019) was also used to conduct the analyses. Supplemental material available at figshare: https://doi.org/10.25387/g3.11774814.

## Results

### Characterization of germplasm

**Figure 1:**
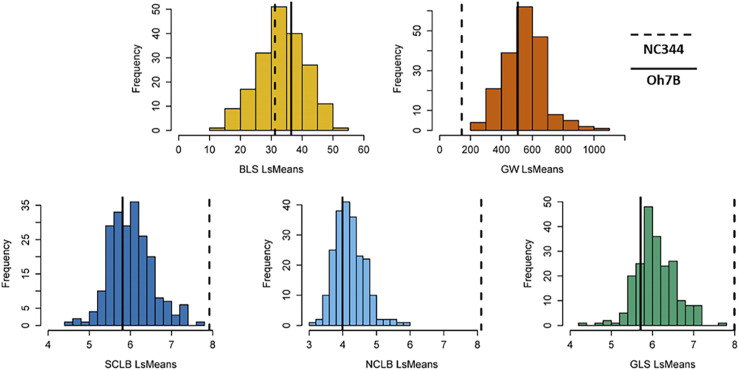
Phenotypic distributions for the DRIL78 (NC344×Oh7B) population for the five traits. The two vertical lines indicate the least square means (LSMeans) of the two parental lines. The recurrent susceptible parent Oh7B is represented by the solid line and the donor resistant parent NC344 is represented by the dashed line. The LSMeans for BLS and GW were based on the lesion length measurement and percent leaf diseased where lower numbers indicate less disease; the LSMeans of SCLB, NCLB and GLS were based on a 1 to 9 scale where lower numbers indicate more disease.

As expected, the recurrent parent Oh7B was the susceptible parent for all the diseases we examined. Of the five diseases, the parents were the most phenotypically similar for BLS. Using a multiple comparison test, we detected significant differences between the donor and recurrent parent for all diseases except BLS. Similar to what has been reported previously for fungal disease phenotypes (Lopez-Zuniga *et al.* 2019), there was substantial transgressive segregation for the bacterial diseases (Figure 1). Like the fungal diseases, the DRIL78 population included lines with transgressive segregation for GW only in the direction of susceptibility, indicating NC344 may donate alleles for both resistance and susceptibility. In contrast, transgressive segregation for BLS occurred in both directions, suggesting that resistance to BLS in NC344 and Oh7B is conditioned by complementary sets of alleles. Using our data, we calculated the heritability for GW: heritability on a plot basis was 0.53 (s.e.= 0.03) and on a family-mean basis was 0.78 (s.e. = 0.02), indicating that progress can be made from inbred line evaluations in breeding for this disease.

Using a multiple test comparison, we examined whether there were DRILs that were significantly more resistant or susceptible than the recurrent parent. For GW, 16 of the 258 lines, or 6.2% of the lines tested, were significantly different than Oh7B (Table 1). Despite the presence of transgressive segregants for susceptibility to BLS, none of the DRILs were significantly more susceptible than Oh7B; however, three lines were significantly more resistant than Oh7B.

**Table 1:** Dunnett’s Multiple Comparison Test for Five Traits in the DRIL78 Population. A Dunnett’s multiple comparison test was conducted to identify lines that were significantly different than the recurrent parent Oh7B. For two disease combinations, all lines were more resistant to both diseases in the combination, except where noted otherwise

Disease	Population Size	Total # of lines significantly different than Oh7B (# more resistant/# more susceptible)
**BLS**	229	3 (3/0)
**GW**	258	16 (3/13)
**SCLB**	216	23 (23/0)
**NCLB**	216	6 (6/0)
**GLS**	216	10 (10/0)
**BLS/NCLB**	189	2
**BLS/GW**	189	1
**SCLB/GLS**	189	1
**SCLB/GW**	189	2*^a^*
**NCLB/GLS**	189	1
**NCLB/GW**	189	2*^b^*
**GLS/GW**	189	1

a Both lines were more resistant to SCLB, but more susceptible to GW.

b Both lines were more resistant to NCLB. Of those, one line was more resistant to GW, while the other was more susceptible to GW.

### Correlation between diseases

We tested pairwise correlations among the five diseases. A total of five of the ten pairwise correlation tests were significant (*P* < 0.05); the two bacterial diseases were not significantly correlated. Of the correlations that were significant, coefficients ranged from 0.15 to 0.31 (Table 2). The correlations for the three fungal diseases vary slightly compared to Lopez-Zuniga *et al.* (2019) as fewer lines are in common for all five diseases as compared to the number of lines in common and included in the correlation analysis for the three fungal diseases. For the three fungal diseases, as previously reported, resistance to NCLB was significantly and positively correlated with resistance to SCLB and GLS, while the correlation between resistance to GLS and SCLB was positive but not significant (Lopez-Zuniga *et al.* 2019). Here, we found significant and positive correlations among pairs of bacterial and fungal diseases (GW and NCLB; GW and GLS; BLS and NCLB). These correlations suggest that loci conditioning MDR to bacterial and fungal diseases may exist in this population, although the correlations could also be due to morphological traits or other factors.

**Table 2:** Pairwise Correlation Coefficients for LSMEANS in the DRIL78 Population. Phenotypic correlations were examined between the five diseases examined in this study

Disease	BLS	SCLB	NCLB	GLS
**GW**	0.12	−0.11	0.31*^a^*	0.24*^a^*
**BLS**		−0.11	0.23*^b^*	0.06
**SCLB**			0.16*^c^*	0.05
**NCLB**				0.15*^c^*

a 0.001 significance level.

b 0.01 significance level.

c 0.05 significance level.

### Identification of multiple disease resistant lines

The correlations between diseases suggested that MDR loci may exist in this population, so we tested whether the same DRILs were significantly more resistant or susceptible than the recurrent parent for multiple diseases. Only 5.3% of the lines (10 of 189 lines) were significantly different than Oh7B for more than one disease. The lines that were significantly different for more than one disease represented seven unique two disease combinations. Not all possible two disease combinations were represented. Only one line was significantly different than Oh7B for the combination of the two bacterial diseases. There were four bacterial/fungal disease combinations, all of which included GW, with seven lines that were significantly more resistant to the combination of a bacterial and fungal pathogen. The remaining two lines were significantly different than Oh7B for a combination of two fungal diseases (SCLB and GLS; NCLB and GLS). For NCLB and SCLB there were lines that were resistant to the respective fungal disease, but susceptible to GW. No lines were significantly different than Oh7B for more than two diseases.

### GW linkage mapping

The genotype and environment interaction accounted for some variance; thus, single environment mapping analysis was also conducted for GW. We conducted linkage mapping for GW for three individual environments, as well as the combined dataset. A total of ten QTL on chromosomes 1 through 6, and 9 were detected (Table 3). Six of the QTL were stable, as they were consistently detected across multiple environments or in the combined dataset. The QTL detected in chromosomal bin 2.07 (*qGW2.07*; peak marker PHM14412-4) was detected in all three individual environments and the combined dataset. The QTL in chromosomal bins 3.06, 4.06 and 9.02 were detected in more than one environment, and the additive effect estimates and percentage of variance explained by these QTL were similar across datasets.

**Table 3:** Significant QTL Detected in DRIL78 Population for GW across all Environments

Peak marker	Chr. *^a^*	cM	Position *^b^*	Bin*^c^*	Environment	LOD*^d^*	Add*^e^*	PVE(%)*^f^*
PHM12633-15	1	116.2	103,835,578	1.05	Combined	3.69	53.72	4.84
PHM14412-4	2	127.4	203,610,640	2.07	Combined	6.58	−63.65	8.96
					Urbana 2016	3.36	−71.34	6.16
					Urbana 2017	3.57	−55.10	5.39
					Monmouth 2017	4.40	−61.68	6.62
PZA00348-11	3	68.94	32,780,891	3.04	Combined	3.38	49.76	4.42
PHM5502-31	3	78.21	68,060,067	3.04	Monmouth 2017	3.32	65.67	5.00
PHM1959-26	3	105.64	170,153,721	3.06	Urbana 2016	4.26	−82.89	7.90
					Monmouth 2017	5.77	−79.25	8.84
PHM15864-8	4	87.18	151,565,558	4.06	Combined	2.83	57.70	3.73
					Urbana 2017	3.28	74.74	5.02
PZA03092-7	5	64.27	12,049,611	5.02	Urbana 2016	3.24	−91.96	5.99
PHM5529-4	6	126.27	167,219,234	6.08	Urbana 2017	4.83	56.39	4.83
PHM5185-13	9	47.48	18,905,238	9.02	Combined	4.76	−72.02	6.38
					Monmouth 2017	3.22	−73.97	4.81
PZA00588-2	9	61.08	62,366,576	9.03	Urbana 2017	5.52	−74.93	8.54

a Chromosome.

b The physical position (RefGen_v3) of significant markers.

c Chromosomal bin location of significant QTL (Davis *et al.* 1999).

d LOD value at the position of the peak likelihood of the QTL. A permutation test was conducted to determine the LOD threshold for the significant markers.

e Additive effect estimates of the detected QTL. Effects are in terms of the disease rating scale used. A negative value indicates that the donor allele increases the disease resistance of the line in the population.

f Percentage of the phenotypic variance explained by the detected QTL.

We examined the additive effect estimates and percentage of variance explained by the significant markers. The GW QTL were of small effect, with the largest-effect QTL, referred to as *qGW2.07*, accounting for 8.96% of the phenotypic variation in the combined dataset. The other QTL explained from 3.73 to 8.84% of the phenotypic variance. The QTL detected on chromosomes 2, 3 and 9 had negative additive effect estimates, indicating that the NC344 allele confers resistance. The QTL with positive additive effect estimates on chromosome 1, 3, 4 and 6 indicate that the Oh7B allele confers resistance. On chromosome 3, two QTL were identified within the same bin. NC344 conferred the resistant allele for both QTL on bin 3.04.

### Multivariate multiple disease mapping

**Figure 2:**
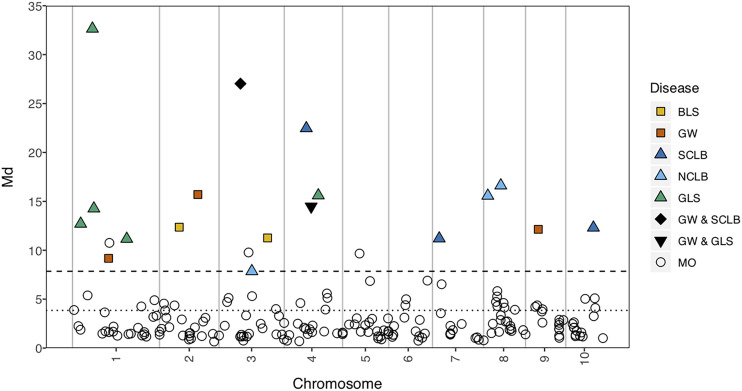
Manhattan plot for multivariate analysis. The mapping results for the two bacterial diseases are represented with warm colors and the three fungal diseases in cold colors. The GW&SCLB and GW&GLS symbols indicate that the same SNP is significantly associated with both diseases. The MO symbol corresponds to the markers that were not significant in the single-trait mapping analysis but were significant in the multi-trait composite analysis. The dotted line indicates the 1% FDR for the Md statistic. The dashed line represents the Md value for the minimum LOD threshold for the five mapping analyses.

Across all diseases, we detected 18 significant markers in the single-trait mapping, with two markers for BLS, five for GW, four for SCLB, three for NCLB and six for GLS. The markers detected in the single-trait mapping were designated “single-trait markers.” Among the 18 single-trait markers, two were shared by multiple diseases (GW and SCLB; GW, and GLS) (Figure 2). Across the single-trait analyses, chromosomes 1 through 4 were all associated with more than one disease (Figure 2).

Multivariate analysis was conducted to detect MDR regions using the robust Mahalanobis distance method (Rousseeuw 1985; Rousseeuw and Van Zomeren 1990). At a 1% false discovery rate, 54 out of 237 markers were detected as related to one or more diseases. The 54 significant markers included all 18 single-trait markers. Several regions emerged as likely MDR candidates. We identified the largest number of significant markers on chromosomes 1 (10 significant markers), 3 (8 significant markers), and 8 (9 significant markers). On chromosome 4, 6 and 10, several markers exceeded the multi-trait threshold, indicating that even markers with relatively low LOD scores for individual diseases can have a high multi-trait Md value (Lopez-Zuniga *et al.* 2019). We observed four co-localized QTL in bin 8.03 and three in bin 9.02. The two regions with markers that were identified for more than one disease in the single trait analysis, specifically bin 3.04 (GW and SCLB) and bin 4.06 (GW and GLS), were also detected in the Md test. In all, five regions with the strongest statistical support and that have been examined in previous studies, were selected to examine their role in resistance to multiple diseases.

### Haplotype effect analysis

**Figure 3:**
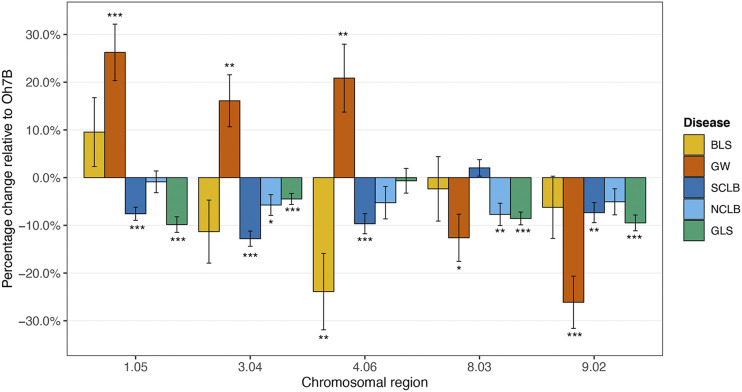
Estimation of haplotype effect. The x-axis indicates the selected genomic regions, and the y-axis indicated the percentage change of disease severity of lines with an introgression at that region. The negative percentage value indicates that lines with an introgression in this region were more resistant than Oh7B and a positive value indicates that the lines were more susceptible. A *t*-test was conducted to examine the significance of bin effect. * indicates the 0.05 significance level; ** indicates the 0.01 significance level and *** indicates that p-value was smaller than 0.001.

We hypothesized that some haplotypes may have opposite effects on bacterial and fungal diseases, *e.g.*, a region may confer resistance to a fungal disease but susceptibility to a bacterial disease. We selected MDR regions located in bins 1.05, 3.04, 4.06, 8.03 and 9.02 to test this hypothesis. We estimated the effect of the haplotype at each of the selected regions, referred to as the haplotype effect, on disease severity for each of the diseases (Figure 3). The MDR region at bin 8.03 was associated with resistance to GW, NCLB and GLS; bin 9.02 was associated with resistance to GW, SCLB and GLS. While the introgressions conditioned resistance relative to Oh7B for these two bins, the effect sizes varied. These may be examples of uneven pleiotropy, whereby an MDR locus has varying effect sizes for different diseases (Wiesner-Hanks and Nelson 2016), or tight linkage. Some regions conferred contrasting effects for the diseases examined: the haplotypes at bins 1.05, 3.04 and 4.06 had an opposite effect for GW as compared to the other diseases. The NC344 haplotype at bin 1.05 was associated with resistance to SCLB and GLS, but susceptibility to GW. The introgressions in bin 3.04 conferred resistance to all the three fungal diseases, but susceptibility to GW. Lines with introgressions at bin 4.06 were more resistant to BLS and SCLB, but more susceptible to GW as compared to Oh7B. The examination of individual loci showed that the same region can confer opposite effects for different diseases and suggests that multiple disease resistance may be linked in these cases, rather than pleiotropic.

## Discussion

The heritability of GW resistance in this population was relatively high and on par with previous GW studies (Ngong-Nassah 1992; Singh *et al.* 2016; Cooper *et al.* 2018). High heritability has been reported for the three fungal diseases for this population, namely 0.76 for SCLB, 0.75 for NCLB, and 0.59 for GLS (Lopez-Zuniga *et al.* 2019), indicating that progress can be made from inbred line evaluations in breeding for these diseases. Bacterial leaf streak had the lowest heritability of the diseases examined in this population: 0.42 (Qiu *et al.* 2020). The GW QTL we identified were relatively stable across multiple environments in the single trait analysis. The QTL in bins 1.05, 2.07 and 9.02 were consistently detected and colocalized with previously identified QTL (Cooper *et al.* 2018; Singh *et al.* 2016).

A central objective of this study was to investigate the relationship between resistance for multiple diseases in a mapping population. Previous studies demonstrated that resistance for the three fungal diseases, namely SCLB, NCLB, and GLS, are correlated with each other. For instance, high positive (>0.5) genetic correlations were detected in a diversity panel between resistance to all the pairwise fungal disease combinations in 253 inbred maize lines (Wisser *et al.* 2011). The DRIL78 correlations for the fungal diseases are not as strong compared to other populations, as no correlation was detected between resistance to SCLB and GLS (Lopez-Zuniga *et al.* 2019). Resistance between these two diseases are typically significantly and highly correlated (Zwonitzer *et al.* 2010). The lack of correlation in this population is likely due to the alleles segregating in this population. We previously reported a significant positive correlation between resistance to a bacterial (GW) and a fungal disease (NCLB) in a different population (Cooper *et al.* 2018). The significant correlations among diseases indicate the possibility of MDR in this population.

Despite the differences between fungal and bacterial pathogens, some of the pathogens can infect the same tissue types, specifically the vasculature. SCLB and GLS are non-vascular diseases (Beckman and Payne 1982; Minker *et al.* 2018), while GW and NCLB are vascular diseases (Minker *et al.* 2018; Mbofung *et al.* 2016). Only one vascular/vascular (NCLB and GW) disease correlation combination was identified. Most combinations were of a vascular and non-vascular disease (NCLB with BLS, NCLB with SCLB, NCLB with GLS, and GLS with GW), indicating that either resistance is linked but not pleiotropic or that there is another resistance mechanism at play that does not interfere with the pathogen’s growth within specific plant tissues.

We found evidence of regions conferring resistance to more than one disease from the single disease analysis. This is consistent with previous reports across multiple species of clustering of regions conferring disease resistance (Wisser *et al.* 2005; McMullen and Simcox 1995; Miklas *et al.* 2000). The same marker was effective for two disease combinations, specifically for the combination of GW and SCLB in bin 3.04 and for the combination of GW and GLS in bin 4.06. This is consistent with the multiple comparison test, where lines effective against these two disease combinations were identified. The Pearson’s product correlation coefficients were significant for the combination of GW and GLS. Interestingly, in both instances, the QTL protect against a combination of a vascular bacterial disease and a non-vascular fungal disease.

To examine MDR in the DRIL78 population, multi-disease post-mapping analysis based on Md was conducted. All 18 of the markers detected in the single-trait mapping analysis were significant in the Md analysis. One possible explanation for this is that significant Md values can arise only due to one trait so that if a marker was highly significant for one disease, it would be identified as an MDR marker as well. The fundamental idea of the Md approach is to identify outliers in multivariate space, and outliers can occur in any one of the dimensions (the five disease-trait dimensions in our case). For the 36 novel markers from the multivariate analysis, LOD values were not high enough to exceed the LOD threshold in the single-trait mapping analysis. However, when combining the five diseases together, creating a new variable Md-p, and testing for Md-p outliers, led to the identification of the additional markers. Lopez-Zuniga *et al.* (2019) also noted this phenomenon when testing for MDR markers using an Md approach.

We found that disease-associated QTL were distributed across all 10 chromosomes, but the QTL were not evenly distributed. This is consistent with previous synthesis studies on the genomic distribution of disease QTL in maize (Wisser *et al.* 2006). Based on the distribution of the single-trait and multi-trait QTL, we focused on five MDR regions to investigate further. Of these five regions, bins 1.05, 3.04, 8.03 and 9.02 have been reported previously to be related to multiple diseases in other populations (McMullen and Simcox 1995; Wisser *et al.* 2006; Ali *et al.* 2013; Lopez-Zuniga *et al.* 2019; Cooper *et al.* 2018). Lopez-Zuniga *et al.* (2019) identified bin 1.05 for resistance to SCLB, NCLB and GLS, and bin 3.04 for SCLB and GLS. Another study in maize utilizing near-isogenic lines found that bin 3.03-3.04 and bin 9.02-9.03 were associated with SCLB, NCLB and GLS resistance (Belcher *et al.* 2012). In addition to the three selected fungal diseases, bin 3.04 was also found to harbor QTL conferring resistance to European corn borer, *Fusarium* stalk rot, common rust and maize mosaic diseases (McMullen and Simcox 1995).

We hypothesized that allele effect sizes differed at each locus for each disease and that some QTL had contrasting effects for different diseases. We found that some regions were associated with resistance to one disease and susceptibility to another, which is consistent with previous findings in other studies (Belcher *et al.* 2012). The introduction of resistance for one disease might unintentionally introduce susceptibility for a second disease. Fine mapping is required to determine whether the same gene is conferring resistance to one disease and susceptibility to another.

The mechanisms underlying MDR in this population remain elusive. Of the combinations of diseases identified using the mulitple comparison and multivariate tests, there was no clear pattern of pathogen kingdom or pathogenesis process in the combinations observed. Thus, if there is a pleiotropic gene underlying these regions, the mechanism is not obviously associated with pathogen kingdom or the growth of the pathogen in the vasculature. Resistance to all five of the diseases examined here is largely quantitative (Qiu *et al.* 2020; Cooper *et al.* 2019; Wisser *et al.* 2006), and thus it is conceivable that common quantitative disease resistance mechanisms could underlie the observed multiple disease resistance. Several mechanisms have been hypothesized to underlie quantitative disease resistance (Poland *et al.* 2009; Yang *et al.* 2017a) and some of these could have effects across pathogen kindgoms and pathogenesis strategies. It is important to note, that this study does not have the resolution to resolve these QTL to single genes, and it is likely that several of these cases are due to linkage, not pleiotropy. Breakpoint analysis is needed to further dissect these loci.

## Conclusion

In summary, a total of five QTL associated with resistance to GW in the combined-environment mapping study were identified, one of which was consistent across all individual environments and the combined-environment mapping analysis. By combining GW mapping results with published data for NCLB, SCLB, GLS (Lopez-Zuniga *et al.* 2019) and BLS (Qiu *et al.* 2020), we identified genomic regions associated with multiple disease resistance. Two markers were identified in the independent single-trait mapping analysis as conferring effects for two diseases. A total of 36 MDR-related markers were identified in the multivariate analysis. Disease QTL were distributed across all ten chromosomes, and we focused on five regions with QTL clustering. We found strong support for multiple disease resistance QTL at 1.05, 3.04, 4.06, 8.03 and 9.02 across multiple analyses. We found evidence of QTL conferring contrasting effects for different diseases. This work deepens our understanding of multiple disease resistance in maize and the relationship between fungal and bacterial disease resistance.
